# Increases of Lipophilic Antioxidants and Anticancer Activity of Coix Seed Fermented by *Monascus purpureus*

**DOI:** 10.3390/foods10030566

**Published:** 2021-03-09

**Authors:** Haiying Zeng, Likang Qin, Xiaoyan Liu, Song Miao

**Affiliations:** 1 Key Laboratory of Plant Resource Conservation and Germplasm Innovation in Mountainous Region (Ministry of Education), Collaborative Innovation Center for Mountain Ecology & Agro-Bioengineering (CICMEAB), College of Life Sciences/Institute of Agro-Bioengineering, Guizhou University, Guiyang 550025, China; hyzeng1@gzu.edu.cn; 2 School of Liquor and Food Engineering, Guizhou University, Guiyang 550025, China; 3 Zhongkai University of Agriculture and Engineering, Guangzhou 510000, China; lxyan0344@163.com; 4 Teagasc Food Research Centre, Moorepark, Co. Cork, D15 DY05 Fermoy, Ireland

**Keywords:** coix seed, *Monascus purpureus*, antioxidant, fermentation, HEp2

## Abstract

Lipophilic tocols, γ-oryzanol, and coixenolide in coix seed before and after fermentation by *Monascus purpureus* were determined. Antioxidant and anticancer activities of raw and fermented coix seed were evaluated using free-radical-scavenging assays and polyunsaturated fatty acid oxidation model, and human laryngeal carcinoma cell HEp2, respectively. Compared to the raw seed, the tocols, γ-oryzanol, and coixenolide contents increased approximately 4, 25, and 2 times, respectively, in the fermented coix seed. Especially, γ-tocotrienol and γ-oryzanol reached 72.5 and 655.0 μg/g in the fermented coix seed. The lipophilic extract from fermented coix seed exhibited higher antioxidant activity in scavenging free radicals and inhibiting lipid oxidation. The inhibitory concentrations for 50% cell survival (IC_50_) of lipophilic extract from fermented coix seed in inhibiting HEp2 cells decreased by 42%. This study showed that coix seed fermented by *M. purpureus* increased free and readily bioavailable lipophilic antioxidants and anticancer activity. Therefore, fermentation could enhance the efficacy of the health promoting function of coix seeds.

## 1. Introduction

Coix (*Coix lacryma-jobi* L. var. adlay) is a cereal widely cultivated in Asian countries, including China, Japan, Thailand, Myanmar, Laos, and India [1]. It has been recommended as a nourishing whole food, since coix seeds contain different amino acids, fibers, and phytochemical antioxidants, especially the lipophilic antioxidant vitamin E (tocols) and γ-oryzanol [2]. Numerous studies have reported different health benefits of the consumption of coix seed including decreasing in low-density lipoprotein cholesterol and increasing high-density lipoprotein cholesterol triglycerides, preventing fatty liver, reducing cell inflammation induced by lipoprotein oxidation, inhibiting allergic effects, etc. [3,4,5]. It has also been used for rheumatism, neuralgia, and diuretic medications and anticancer treatment [1,6]. However, those previous studies mainly focused on raw coix seed. As most of the antioxidants in cereals are in bound or blocked form due to cellulose, they are not readily converted to a free form in normal cooking processes and in the human digestive tract [7]. Thus, the bioavailability and bio-absorption of the bound-form antioxidants in the body are usually much lower than that of the free-form antioxidants.

Recently, the fermentation by probiotic microorganisms as a pre-digestion process has been applied to release the bindings of bound hydrophilic antioxidants and increase free-form antioxidants and their bioavailability [8]. However, the effects of the fermentation process on bound lipophilic antioxidants in cereal have not been reported. In this study, the changes in lipophilic antioxidants and the antioxidant and anticancer activity of coix seed fermented by *Monascus purpureus* were evaluated. The increase in the levels of free-form antioxidants by fermentation could enhance their bioavailability or bio-absorption in the body and increase the efficacy of the health-promoting functions of coix seed.

*M. purpureus* is a fungus, traditionally used in the preparation of fermented grains in China, such as fermented rice [9,10]. The fermentation process increases organoleptic qualities including desirable pigment and flavor. It also inhibits the growth of pathogenic microorganisms by producing organic acids and other compounds, thereby extending the shelf-life of fermented rice [11]. Previous studies found that the antioxidants of sweet potato, oat, and soybean, especially hydrophilic antioxidants, such as total phenolics and flavonoids, were significantly increased due to fermentation of *Lactobacillus acidophilus, Aspergillus oryzae, Monascus anka,* and *Bacillus subtilis* [8,9,12]. The results showed that fermentation was one of the effective and practical ways to increase the hydrophilic antioxidants in those plant foods. Fermentation not only assists in releasing those bound antioxidants to increase their bioavailability, but it could also reduce the sugar level in fermented foods. The fermented food can also help people with small-intestinal bowel infection or colonic infection effectively metabolize and absorb those antioxidants in the fiber-rich cereals [8].

In this study, the antioxidant activities of the lipophilic extracts from raw and fermented coix seed were measured using DPPH (2,2-diphenyl-1-picrylhydrazyl), ABTS (2,2′-azino-bis(3-ethylbenzothiazoline-6-sulfonic acid)) cation, and superoxide anion radical-scavenging assays. The activities of the extracts from raw and fermented coix seed in stabilizing susceptible polyunsaturated fatty acids C20:5 and C22:6 were evaluated as well. As vitamin E is an important lipophilic antioxidant in the human body, the anticancer activities of lipophilic extracts from raw and fermented coix seed were determined using HEp2 laryngeal carcinomatous cells. The results of this study could be helpful in understanding the changes of lipophilic antioxidants in fermented cereals. The fermented cereal or its extract could be used as a healthy processed food or ingredient with highly bioavailable bioactivity and enhanced health promoting function.

## 2. Materials and Methods

### 2.1. Chemicals and Materials

HPLC-grade hexane and acetic acid were purchased from Fisher Chemicals (Fair Lawn, NJ, USA). α- and γ-Tocopherols, α- and γ-tocotrienols, γ-oryzanol, 2,2-diphenyl-1-picrylhydrazyl (DPPH), 2,2′-azinobis(3-ethylbenzothiazoline-6-sulfonic acid)(ABTS), EDTA, Trolox, Tween 20, and menhaden fish oil were purchased from Sigma Aldrich (St. Louis, MO, USA). Human laryngeal carcinoma cell HEp2 and normal monkey kidney cell CV-1 lines were purchased from American Type Culture Collection (ATCC, Manassas, VA, USA). Other cell culture reagents, Dulbecco’s modified Eagle medium (DMEM), fetal bovine serum (FBS), antibiotic (penicillin–streptomycin), phosphate-buffered saline (PBS), dimethyl sulfoxide (DMSO), and CellTiter-Blue were purchased from Invitrogen (Grand Island, NY, USA). *M. purpureus* (CGMCC 3.4629) was purchased from China General Microbiological Culture Collection Center (Beijing, China). Broken coix seed was provided by Xinlong Green Development Company (Guizhou, China).

### 2.2. Fermentation of Coix Seed Using M. purpureus Strain

The *M. purpureus* strain was cultivated on potato dextrose agar (PDA) medium at 30 °C for 7 days. The spores were harvested in sterile distilled water and then adjusted to prepare a solution with concentration of 10^6^ spores/mL. Coix seed were sterilized at 121 °C and 15 psi for 20 min. Then, the strain solution was mixed with the sterilized seed at a ratio of 1:10 (v/w). The mixture was incubated at 30 °C for 10 days. The pigment extraction and estimation were performed according to the method described by Marič [10]. The fermentation was independently carried out in triplicate.

### 2.3. Extraction and Determination of Lipophilic Antioxidants and Coixenolide in Raw and Fermented Coix Seed

Lipids in the seed were extracted by the method described by Shen with slight modifications [13]. Briefly, raw or fermented coix seed (10 g) was extracted using 50 mL of hexane at 45 °C for 20 min. The extraction was repeated three times. All collected supernatants were combined and evaporated using a vacuum centrifugal evaporator (CentriVap Mobile System; Labconco, Kansas City, MO, USA) to remove the hexane solvent. The dried extract was weighed (dry weight basis) and dissolved in isopropanol to prepare a stock solution (100 mg/mL).

Tocopherols, tocotrienols, and oryzanol in the extract were determined using a normal-phase HPLC system (1100 series; Agilent, Santa Clara, CA, USA) with Supelcosil LC-Si column (id 250 × 4.60 mm 5 μm, Supelco, Bellefonte, PA, USA) and a series of fluorescence and UV detectors. The HPLC analysis conditions were as described by Jang and Xu [14]. The concentration of each component was calculated based on standard curves. Coixenolide was determined by the method of Yang et al. [15].

### 2.4. Determination of Free-Radical-Scavenging Activity of the Lipophilic Extract

During fermentation, the DPPH-scavenging activity of lipophilic extract from fermented seed was determined every day. The DPPH-scavenging activity of Trolox was used to prepare a calibration curve of the activity. The DPPH-scavenging activity of lipophilic extract was calculated and converted to μmol Trolox equivalent/gram.

The DPPH assay was performed according to a previous study with slight modifications [16]. Briefly, 1.9 mL of methanolic solution containing 0.1 mM DPPH radicals was mixed with 0.1 mL of different concentrations of sample solutions in a range of 0 to 100 mg/mL and/or methanol used as a blank. The mixtures were vortexed thoroughly and then stood in a dark area for 30 min at room temperature. The absorbance of the reaction mixture was measured at 517 nm using a spectrophotometer. The DPPH free-radical-scavenging activities were calculated as follows:

DPPH free-radical-scavenging rate (%) = (1 − Abs_sample_/Abs_blank_) × 100 (1)

where Abs_sample_ and Abs_blank_ were the absorbance of the mixture of blank and sample with DPPH reagent after reaction, respectively.

For ABTS cation radical-scavenging activity, the measurement method was based on our previous research [4]. Briefly, 9 mL of ABTS solutions was mixed with 3 mL sample solutions with different concentrations (0–100 mg/mL). Methanol was used as a blank. The mixtures were incubated in dark at room temperature for 20 min. The absorbance of each mixture was measured at 734 nm. The ABTS-scavenging activity was calculated according to the equation:

ABTS-scavenging rate (%) = {[Abs_0_ − (Abs_1_ − Abs_2_)]/Abs_0_} × 100 (2)

where Abs_0_ was the absorbance of ABTS solution; Abs_1_ was the absorbance of the reacted mixture of ABTS with tested sample; Abs_2_ was the absorbance of the methanol with tested sample.

The superoxide anion radical-scavenging activity of the extract was measured based on the method in previous study [17]. Different concentrations of the sample solutions (0.1 mL) were mixed with 4.5 mL of 0.05 mol/L Tris-HCl buffer (pH 8.2) containing 2 mmol/L EDTA. The mixtures were reacted at 25 °C for 20 min. Then 0.4 mL of 25 mmol/L 1,2,3-phentriol was added and allowed to react at 25 °C for 5 min more. Finally, to the mixture was added 1 mL of HCl (8 mol/L) to stop the reaction. The absorbance was recorded at 325 nm. Methanol was used as a blank. The superoxide anion radical-scavenging activity was calculated as follows:

Superoxide anion radical-scavenging rate (%) = (1 − Abs_sample_/Abs_blank_) × 100 (3)

where Abs_blank_ was the absorbance of the control group and Abs_sample_ was the absorbance of mixtures with sample solutions.

### 2.5. Determination of Anti-Lipid-Oxidation Activity Using Fatty Acid Model

The anti-lipid-oxidation activity of lipophilic extract from raw or fermented seed was determined according to a previous study by Zhang et al. [18]. Briefly, 2 mL sample solution (100 mg/mL) was mixed with 20 mL of fish oil emulsion, which consisted of 10 g menhaden fish oil, 10 mL Tween 20, and phosphate buffer. Then, the mixed emulsion was incubated at 37 °C for 5 days with continuously stirring. The concentrations of fatty acid EPA (C20:5) and DHA (C22:6) in the mixed emulsion were measured at day 1, 3, and 5. The fatty acid analysis was performed on a GC system equipped with an FID detector with analysis condition described in the study of Zhang et al. [18]. The emulsion without sample solution was used as a blank control. Anti-lipid-oxidation activities were expressed by the retained rate of DHA and EPA and were calculated as follows:

Retained DHA or EPA rate (%) = (C_t_/C_0_) × 100 (4)

where C_0_ was the original concentration of EPA or DHA; C_t_ was the concentration of EPA or DHA at different incubation time.

### 2.6. Determination of Anticancer Activity of Lipophilic Extract on HEp2 Cells

Anticancer activities were determined based on the survival rate of HEp2 cells treated with different concentrations of lipophilic extract from raw or fermented coix seed. The procedure was described in our previous study [13]. The cells were maintained in 95% DMEM (containing 10% FBS and 1% penicillin−streptomycin) and incubated with 5% CO_2_ at 37 °C. The cells were placed in a 96-well plate and incubated for 24 h. Then, the cells were exposed to the medium with different concentrations (0 as blank, 0.625, 1.25, 2.5, 5, and 10 mg/mL) of lipophilic extract from coix seed and incubated for another 24 h at 37 °C. After incubation, the cells were washed three times with PBS and mixed with new medium containing 20% CellTiter-Blue. They were incubated at 37 °C for 4 h. By using a FluoStar Optima microplate reader, the cell viability or survival rate in each well was determined at excitation and emission wavelengths of 570 and 615 nm, respectively. Meanwhile, the normal monkey kidney cells, CV-1, were used as the control, and were treated with the same concentration extract of fermented coix seed. The survival rate of each concentration’s treatment group relative to that of the blank group was used to express anticancer activity.

### 2.7. Data Analysis

The determinations of lipophilic antioxidants and the evaluations of antioxidation activities were carried out in triplicate and expressed as mean with standard deviation values. The SPSS 22.0 software (IBM Company, New York, NY, USA) was used for statistical analysis. The significant differences between the two groups were determined by ANOVA (SAS, 9.1.3, Cary, NY, USA). Difference between two groups was determined at a significant difference *p* < 0.05 or at an extremely significant difference *p* < 0.01. The determinations of anticancer activities of extracts were repeated five times and analyzed by GraphPad Prism (version 6.0; GraphPad Software Inc., La Jolla, CA, USA).

## 3. Results and Discussion

### 3.1. Lipophilic Antioxidants and Coixenolide in Raw and Fermented Coix Seed

**Figure 1:**
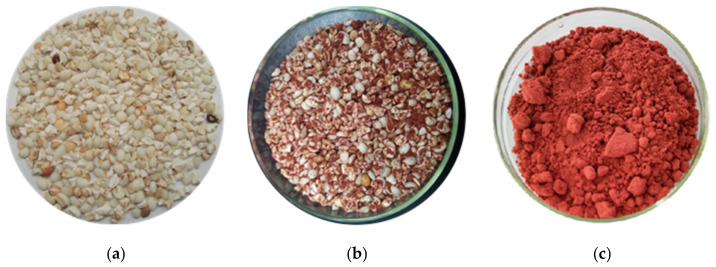
Images of raw coix seeds (**a**), coix seeds in the middle of fermentation (**b**), and coix seed powder after fermentation (**c**).

Figure 1 shows the color changes of coix seeds at different fermentation times. The original color of raw coix seeds was yellowish white and changed to a brownish color in the middle of fermentation (Figure 1a,b). Eventually, the fermented coix seeds had reddish brown color and were readily crushed to a fine powder (Figure 1c). At this time, color value units (CVUs) of pigments in fermented coix seeds reached 687.2 CVU/g. Usually, the growth of *Monascus* sp. and the change of substrate fermentation could be preliminarily judged by the accumulation of pigments. The *Monascus* pigments were the secondary metabolites of polyketides and were biosynthesized by malonyl-CoA catalysis from tetraketide and pentaketide to hexaketide [10,19]. They were accumulated in the solid-state aerobic fermentation of *Monascus* sp.

The yield of lipophilic extract from raw coix seeds was 4.2%, while it increased to 8.6% from fermented coix seeds after 10 days of fermentation. The fermentation significantly released bound-form lipophilic compounds in coix seeds, which were likely contributed by enzymatic hydrolysis induced by *M. purpureus* during fermentation [20]. As fermentation could produce a significant amount of acid and lower pH in the fermented medium, acid hydrolysis would also contribute to the release of bound or blocked forms of lipophilic compounds [8].

**Figure 2:**
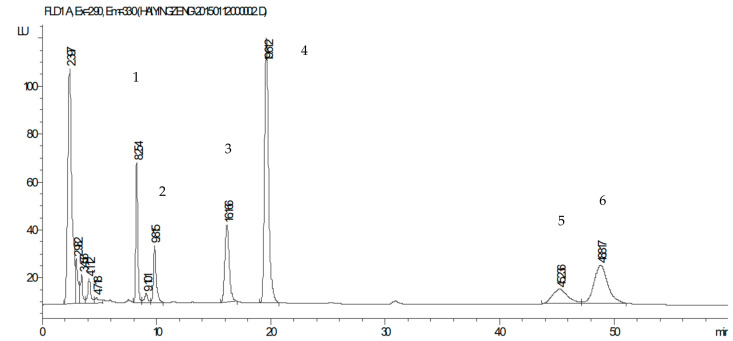
HPLC chromatogram of lipophilic extract from fermented coix seeds: (1) α-tocopherol, (2) α-tocotrienol, (3) γ-tocopherol, (4) γ-tocotrienol, (5 and 6) γ-oryzanols.

Four tocols—α-tocopherol, α-tocotrienol, γ-tocopherol, and γ-tocotrienol—were found in both raw and fermented coix seeds (Table 1 and Figure 2). Although vitamin E has eight different tocols—α, β, γ, and δ-tocopherols and α, β, γ, and δ-tocotrienols, α-tocopherol, α-tocotrienol, and γ-tocopherol are the common tocols in most cereals, beans, and grains [21]. In raw coix seeds, γ-tocopherol was the leading tocol at a level of 21.2 μg/g DW, followed by γ-tocotrienol, while α-tocopherol and tocotrienol were at a very low level in raw coix seeds (Table 1). Typically, α-tocopherol and α-tocotrienol were the dominant tocols in beans and grain oils, followed by γ-tocopherol. Therefore, the profile of tocols in coix seeds was different from that of most cereals, but similar to rice bran, which contains a high level of γ-tocotrienol [14]. After fermentation, α-tocopherol and γ-tocotrienol were significantly increased (*p <* 0.01), approximately 160 (from 0.1 to 17.9 μg/g) and 16 (from 4.4 to 72.5 μg/g) times, respectively (Table 1). It indicated that raw coix seeds had a high level of the bound or blocked form α-tocopherol and γ-tocotrienol. γ-Tocotrienol recently has been reported to have greater health promoting function than other tocols [22,23]. Therefore, the high level of γ-tocotrienol in coix seeds fermented by *M. purpureus* could significantly enhance the health benefits of coix seeds.

The level of γ-oryzanol in fermented coix seeds increased about 25 times to 655.0 μg/g from the level of 26.2 μg/g in raw coix seeds (Table 1 and Figure 2). γ-Oryzanol is usually rich in rice bran and not commonly present in most cereals. It is a type of plant sterol and contains ferulic acid in its structure [24]. Thus, γ-oryzanol is an antioxidant phytosterol because ferulic acid is a strong antioxidant phenolic. It has a potent antioxidant activity in preventing cholesterol oxidation, which could generate toxic cholesterol oxidation products in foods and human body, resulting in development of cardiovascular diseases [25]. Although the level of γ-oryzanol in raw coix seeds was much lower compared to that of most rice varieties (26.2 vs. 200–300 μg/g), the level in fermented coix seeds (655.0 μg/g) was much higher than the level in rice [26]. It was reported that rice bran fermentation by *Rhizopus oryzae* could also increase the free γ-oryzanol level but only by 1.5 times that of raw rice bran. [27]. During fermentation, the microorganisms could degrade the plant cell wall through a variety of self-generated enzymes that stimulate the release of intracellular compounds, especially fiber-bound compounds [28].

**Figure 3:**
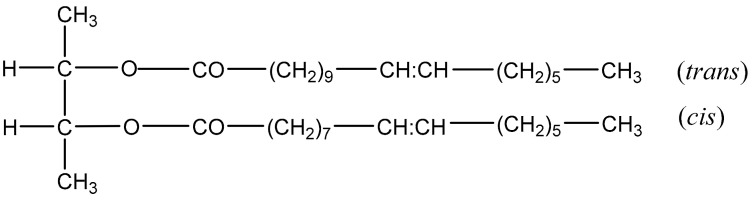
Chemical structure formula of coixenolide in coix seeds (C_38_H_70_O_4_).

Coixenolide is a diol lipid uniquely present in coix seeds. It is a long carbon chain with two long-chain fatty acid esters (Figure 3). Although it may not have antioxidant function based on its chemical structure, previous studies reported that it has outstanding anticancer activity [1,6]. After the fermentation by *M. purpureus*, coixenolide in fermented coix seed increased to 8.7 mg/g, which was about 2.2 times higher than that in raw coix seed (Table 1). The increase of coixenolide could assist in the enhancement of health benefits of coix seeds.

### 3.2. Scavenging Activities on Different Free Radicals of Lipophilic Extracts from Raw and Fermented Coix Seed

**Figure 4:**
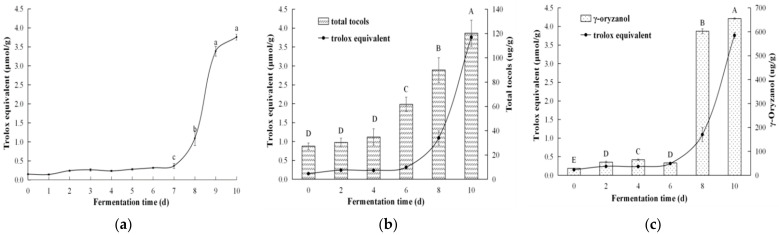
Changes of DPPH-scavenging activity (**a**), total tocols (**b**) and γ-oryzanol (**c**) of lipophilic extract from coix seeds during fermentation. Bars with different letters indicate significant differences (lowercase letters, *p <* 0.05; capital letters, *p <* 0.01).

DPPH-scavenging activity is widely used as an index of antioxidant activity of a test sample in vitro. The activity of lipophilic extract in fermented coix seeds at different fermentation times was monitored. The scavenging activity rose slightly within the first 7 days of fermentation (Figure 4a), then it dramatically increased from 0.4 to 3.3 μmol/g of Trolox equivalent after 9 days of fermentation. The increase of the activity began to slow down from day 9 to day 10 of fermentation. After 10 days of fermentation, the scavenging activity of the lipophilic extract reached 3.8 μmol/g of Trolox equivalent. Tocols and γ-oryzanol were monitored simultaneously during fermentation. The results displayed that the level of total tocols and γ-oryzanol was extremely significantly enhanced during fermentation (*p <* 0.01), especially after the 6th day of fermentation (Figure 4b,c). Correlation analysis showed that total tocols had a significantly positive correlation with DPPH-scavenging activity at a correlation coefficient of 0.9. The correlation coefficient of γ-oryzanol was only 0.8. Thus, tocols could be the major contributor in scavenging DPPH free radicals.

Free radicals are a group of very reactive species that not only cause rancid deterioration in food products but also result in oxidative inflammation in mammalian cells, leading to eventual development of different chronic diseases, such as cardiovascular diseases and cancers [29]. The harmful free-radical initiators can be effectively annihilated by antioxidants to prevent their negative impacts. Antioxidants from natural sources, such as cereals, vegetables, and fruits are considered as safe antioxidants compared to synthetic antioxidant, such as BHA (butylated hydroxyanisole), BHT (butylated hydroxytoluene), and PG (propyl gallate) [30]. Raw and fermented coix seeds with high levels of tocols and γ-oryzanol could be a rich source of natural antioxidants destined for use as ingredients in food products.

**Figure 5:**
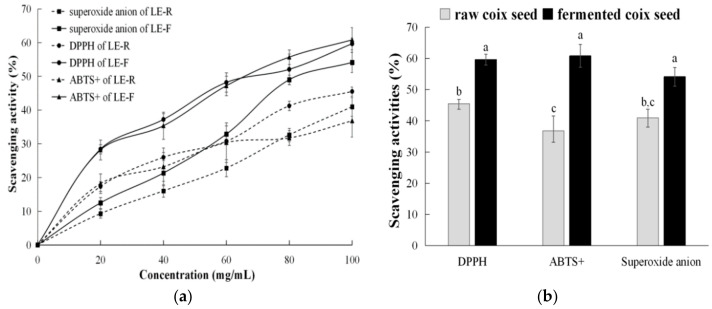
DPPH, ABST cation, and superoxide anion free-radical-scavenging activities of lipophilic extracts from raw and fermented coix seeds at 0–100 mg/mL (**a**) and at 100 mg/mL (**b**). LE-R, lipophilic extract from raw coix seeds. LE-F, lipophilic extract from fermented coix seeds.

Figure 5a shows the scavenging activities of lipophilic extracts from raw and fermented coix seeds at 0–100 mg/mL. The scavenging activities increased with the concentration increase of lipophilic extracts. It also revealed dose–response relationships between the additive concentration of extracts and scavenging activities. The extract from fermented coix seeds on scavenging DPPH, ABTS cation, or superoxide anion free radicals was significantly higher than that of the extract from raw coix seeds at 100 mg/mL (Figure 5b). Moreover, in three different scavenging tests, the lipophilic extract of fermented coix seeds had the most prominent scavenging effect on ABTS cation. The scavenging activity (60.79%) increased 1.7 times after fermentation, followed by superoxide anion and DPPH (about 1.3 times). The effective concentration for 50% of scavenging DPPH rate of pure α-tocopherol is 120 μmol/L or 51.6 μg/mL [30]. α-Tocopherol was normally considered to be the most active tocol in scavenging different free radicals and had 1.5 times higher activity than γ-tocopherol [31]. The increased α-tocopherol in fermented coix seeds could be an important factor for the higher free-radical-scavenging activity, in addition to γ-tocotrienol and γ-oryzanol.

Traditionally, the antioxidant activity of a compound is assessed by measuring its scavenging activity against DPPH or other free radicals and through reducing power assays [32]. However, a good result in these chemical-based spectrophotometric assays cannot predict whether the sample has a good antioxidant capability in inhibiting lipid oxidation in an emulsion. In other words, the higher free-radical-scavenging activity of an antioxidant may not be closely correlated with the actual antioxidant activity in inhibiting the lipid oxidation in a lipid-rich food or oxidative inflammation in mammalian cell in which fluid is a complicated emulsion [30]. Therefore, the antioxidant activity of the lipophilic extract of fermented coix seeds was further evaluated using an oil emulsion model.

### 3.3. Anti-Lipid-Oxidation Activities of Lipophilic Extracts from Raw and Fermented Coix Seeds

In this study, fatty acids, the key component of lipids, were used as the substrates to evaluate antioxidant activity of lipophilic extracts from coix seeds. Compared with free-radical-scavenging chemical assays, the activity obtained in this method is closely correlated to the capability of the antioxidant in stabilizing lipids in a food matrix to extend its shelf life or in a biological system to prevent oxidative stress [18,30]. EPA (C20:5) and DHA (C22:6), polyunsaturated long-chain fatty acids, are the most vulnerable fatty acids to oxidation. Fatty acids could be oxidized to produce a group of short-chain aldehydes, alcohols, and other components. Therefore, the retention of EPA and DHA in oil emulsion with or without the lipophilic extract from coix seeds was monitored. The retained rates of these susceptible polyunsaturated fatty acids could be more accurate to indicate the oxidation status in the emulsion [30]. For example, although α-tocopherol was considered to be the most active tocopherol in reacting with free radicals, in a liposomal membrane model, tocotrienols had higher antioxidant activity than tocopherols [23]. In a recent study, the activity of γ-tocotrienol was also higher than that of α-tocopherol in oil systems [31].

**Figure 6:**
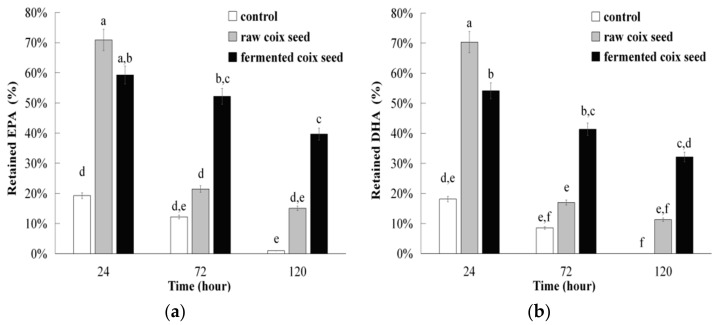
Retention rates of EPA (20:5) (**a**) and DHA (22:6) (**b**) of lipophilic extracts from raw and fermented coix seeds. Bars with different letters indicate significant differences at *p <* 0.05.

The retention rates of EPA and DHA in the control group decreased to below 20% in 24 h and almost totally oxidized after 120 h (Figure 6a,b). Both of the lipophilic extracts from raw and fermented coix seeds still had much higher retention rate for EPA or DHA after 120 h of incubation. Although the extract from fermented raw coix seeds had slightly lower retention rate of EPA or DHA than the extract from raw coix seeds at 24 h, its retention rate at 72 and 120 h of incubation was more than two times higher than that of the extract from raw coix seeds (Figure 6a,b). It still retained approximately 40% and 32% for EPA or DHA after 120 h of incubation, respectively (raw coix seed, only 15% and 11%). The results were similar to that in a previous study that reported anti-lipid-oxidation activity of tocols extract from rice bran against EPA and DHA oxidation [18]. Overall, the results indicated that fermented coix seeds had higher anti-lipid-oxidation activity than raw coix seeds in inhibiting lipid oxidation. Recently, γ-oryzanol as a new lipophilic antioxidant has been studied to stabilize corn oil and fish oil in yogurt [33,34]. Fermented coix seeds could be a good food preservative to reduce lipid oxidation in food. It could also have a higher antioxidant function to lower oxidative stress in a biological system.

### 3.4. Anticancer Activities of Lipophilic Extracts from Raw and Fermented Coix Seeds

**Figure 7:**
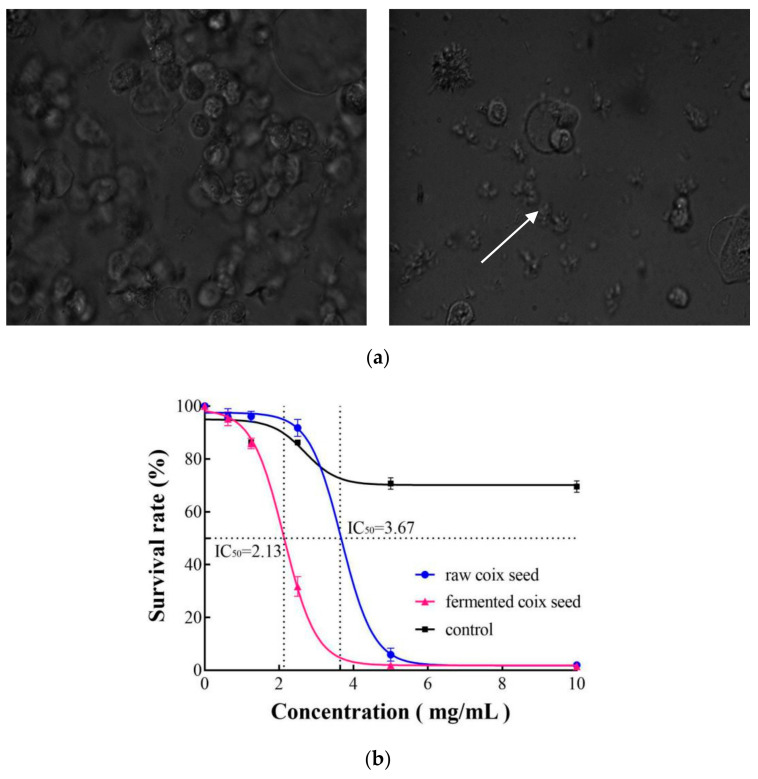
Images of HEp2 cell morphology in medium in the control (**a-left**) and treatment (**a-right**) groups, and survival rates of HEp2 cells incubated with lipophilic extracts from raw and fermented coix seeds (**b**).

The anticancer activities of raw and fermented coix seed were evaluated by using the HEp2 cell line, which is a human laryngeal carcinomatous cell. Laryngeal carcinoma is one of the most common respiratory cancers and accounts for 25% of head and neck carcinomas [35]. Figure 7a shows the images of HEp2 cell morphology in control and treatment groups after 24 h of incubation. The inhibitory concentrations for 50% of cell survival rate (IC_50_) of lipophilic extracts from raw and fermented seeds were 3.67 and 2.13 mg/mL, respectively (Figure 7b). The IC_50_ of lipophilic extract from fermented coix seeds in inhibiting HEp2 cell decreased by 42%. The anticancer activity of lipophilic extract was significantly improved after the fermentation, but its cytotoxicity toward normal CV-1 cells was relatively low. Even at the concentration of 10 mg/mL, only 30.5% of the normal cells were inhibited. The IC_50_ values of lipophilic extracts from raw and fermented seeds for HEp2 cells were higher than that of the lipophilic extract from butterfly pea seeds, for which the IC_50_ for HEp2 cells is 8 mg/mL [13]. In a previous study that compared anticancer activity for T24 cell, a human urinary bladder cancer cell, of coix seed oil extracts from 11 different varieties, all of them had IC_50_ values higher than 0.33% or 3.3 mg/mL in medium [6]. Thus, the anticancer activity of coix seeds could be improved through the fermentation by *M. purpureus*.

Tocols and γ-oryzanol could be the primary compounds responsible for the anticancer activity of the lipophilic extract from coix seeds, besides coixenolide. For example, tocols are involved in the DR5 (death receptor 5) protein upregulation, which stimulates tumor necrosis and restricts its proliferation [36]. For antioxidant phytosterols, such as γ-oryzanol, they could inhibit cancer cell proliferation by meddling with protein phosphatase 2A (PP2A) in the sphingomyelin cycle and blocking the cell cycle at G0/G1 phase in different cancer cells, such as prostate cancer, hepatocyte, and breast cancer cells [37,38]. Therefore, the increase of tocols and γ-oryzanol could assist in the improvement of anticancer activity of the lipophilic extract from fermented coix seeds.

## 4. Conclusions

In summary, this study revealed positive changes in lipophilic antioxidants and anticancer activity of coix seeds after fermentation by *Monascus purpureus*, a typical mold used in the preparation of fermented grains. The fermented coix seeds had increased tocols (vitamin E), γ-oryzanol, and coixenolide contents. The levels γ-tocotrienol or γ-oryzanol in fermented coix seeds were much higher than the level found in most cereals. The increased antioxidants enhanced the antioxidant activity in scavenging different free radicals and stabilizing susceptible polyunsaturated fatty acids. The anticancer activity of coix seeds was significantly improved after the fermentation by *M. purpureus*. Therefore, the fermentation by *M. purpureus* is a good approach in increasing the lipophilic bioactivity and bioavailability of health-promoting compounds in cereals. The fermented coix seed or its extract could be used as a food, food ingredient, or nutritional supplement to provide added health benefits to humans.

## 5. Patents

A new product, coix seed tea fermented by *Monascus purpureus*, has been described in China patent no. CN 201810372538.3.

## Figures and Tables

**Table 1:** Lipophilic antioxidants and coixenolide in raw and fermented coix seeds.

Compound	Raw Coix Seed	Fermented Coix Seed
α-Tocopherol (μg/g DW)	0.1 ± 0.1 ^A^	17.9 ± 7.1 ^B^
α-Tocotrienol (μg/g DW)	2.4 ± 0.6 ^A^	4.2 ± 3.5 ^A^
γ-Tocopherol (μg/g DW)	21.2 ± 3.9 ^A^	25.4 ± 3.2 ^A^
γ-Tocotrienol (μg/g DW)	4.4 ± 1.5 ^A^	72.5 ± 10.8 ^B^
Total tocols (μg/g DW)	28.1	120.0
γ-Oryzanol (μg/g DW)	26.2 ± 4.1 ^A^	655.0 ± 30.1 ^B^
Coixenolide (mg/g DW)	4.0 ± 0.2 ^A^	8.7 ± 0.8 ^B^

Difference between data with different letters in a row is extremely statistically different (*p <* 0.01).

## Data Availability

Data is contained within the article.
